# Utilization of an Optimized Radiation Strategy in Primary Percutaneous Coronary Intervention for Patients with ST-Segment-Elevation Myocardial Infarction

**DOI:** 10.1155/2019/6094806

**Published:** 2019-09-02

**Authors:** Xin Zhong, Wei Gao, Dong Huang, Lei Ge, Juying Qian, Junbo Ge

**Affiliations:** ^1^Shanghai Institute of Cardiovascular Diseases, Zhongshan Hospital, Fudan University, Shanghai 200032, China; ^2^Institutes of Biomedical Sciences, Fudan University, Shanghai 200032, China

## Abstract

**Background:**

Recent reports about radiation risk gradually raised the safety concerns for interventional therapy. However, limited data exist on the optimized radiation strategy in primary percutaneous coronary intervention (P-PCI) for patients with ST-segment-elevation myocardial infarction (STEMI).

**Methods:**

A total of 214 STEMI patients undergoing P-PCI were retrospectively analyzed. Patients were divided into the optimized radiation strategy (ORS) group (*N* = 151) and normal radiation strategy (NRS) group (*N* = 63) according to the radiation protocol utilized. The primary endpoint was the relative dose reduction of total air kerma. The secondary endpoint was 30-day major adverse cardiac and cerebrovascular events (MACCE), as a composite of all-cause death, reinfarction, ischemia-driven target vessel revascularization, and stroke.

**Results:**

Patient groups were well matched for baseline characteristics. There were no differences in terms of age, body mass index, radial artery access, nonculprit vessel PCI, and fluoroscopy time between 2 groups. With optimized radiation strategy, a 40.9% radiation dose reduction (901.2 ± 628.7 mGy versus 1524.0 ± 866.6 mGy, *p* < 0.001) was obtained for total air kerma. No significant differences were found for 30-day MACCE between 2 groups (2.0% versus 1.6%, adjusted hazard ratio: 0.7, 95% confidence interval: 0.1 to 8.6, *p*=0.772).

**Conclusion:**

With optimized radiation strategy, significant radiation dose reduction could be achieved in P-PCI for STEMI patients. It appears to be feasible and safe to carry out the optimized radiation strategy in P-PCI for STEMI patients.

## 1. Introduction

At present, timely primary percutaneous coronary intervention (P-PCI) is the best therapeutic strategy for ST-segment-elevation myocardial infarction (STEMI) [1, 2]. Compared with patients scheduled for selective PCI, STEMI patients in P-PCI are at high risk of acute cardiac events. Many critical situations such as tachyarrhythmia, hypoperfusion, and cardiac shock could have certain adverse effects on imaging and judgment for lesions, hence then raise a claim for a good image quality.

Recent reports about radiation risk gradually raised the safety concerns for the ionizing radiation-mediated interventional therapy [3–5]. Although there have been some radiation reduction measures [6–9] reported to reduce radiation dose, all attempts were made for routine coronary angiography or selective PCI. However, as a common sense, radiation exposure reduction would inevitably affect the image quality. Up to now, the evaluation of optimized radiation strategy in P-PCI has never been reported yet.

Recently, we reported the utilization of optimized radiation strategy in chronic total occlusion- (CTO-) PCI [10]. Herein, by evaluating radiation dose parameters and 30-day clinical outcomes in the present study, we investigated for the first time the efficacy and safety of an optimized radiation strategy in P-PCI for STEMI patients.

## 2. Materials and Methods

### 2.1. Study Population

**Figure 1:**
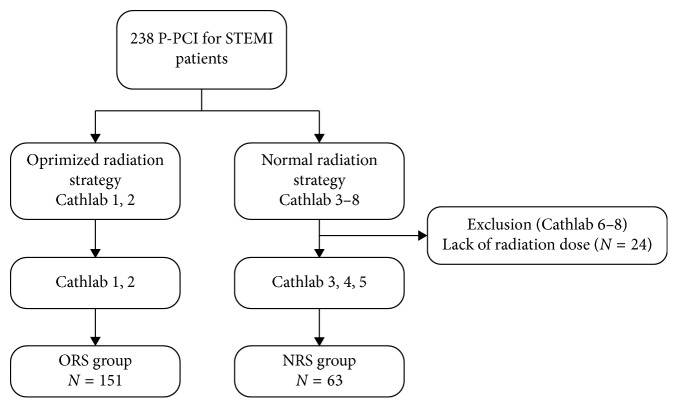
Flow chart of the study. P-PCI, primary percutaneous coronary intervention; STEMI, ST-segment–elevation myocardial infarction; ORS, optimized radiation strategy; NRS, normal radiation strategy.

Between September 2016 and September 2017, all P-PCI for STEMI patients with an onset-to-door time of <24 h were retrospectively screened from database of our intervention center. Unfortunately, due to setting of software, 3 X-ray systems without optimized radiation strategy could not upload the radiation dose value automatically. Therefore, all P-PCI performed within these 3 X-ray systems (*N* = 24) were excluded from the present study. As a result, a total of 214 STEMI patients were included in the present study (Figure 1). All patients were divided into optimized radiation strategy (ORS) group (*N* = 151) and normal radiation strategy (NRS) group (*N* = 63) according to the radiation protocol utilized. During the period of this study, as operators became aware of inter-equipment differences, more P-PCI was carried out following the optimized radiation strategy unless the relevant X-ray systems were occupied or in maintenance. Each P-PCI was performed according to the current guidelines [2] and successful PCI was defined as TIMI grade 2 to 3 flow after P-PCI.

This retrospective study was approved by the medical ethics committee of Zhongshan Hospital (No. B2017-173). All procedures performed in studies involving human participants were in accordance with the ethical standards of the institutional and/or national research committee and with the Helsinki declaration and its later amendments.

### 2.2. Imaging Protocol

The optimized radiation strategy was carried out in two identical X-ray systems (Philips AlluraXper, 2013 manufactured/2014 installed, Royal Philips Electronics, Amsterdam, Netherlands) in our intervention center, of which the radiation parameters set were consistent and synchronized. As we previously reported, the fluoroscopy frame rate was set as 15 f/s and cine-angiography frame rates as 7.5 f/s. On the other hand, a 0.9 mm/1.0 mm copper (Cu)/aluminum (Al) filter was implemented for fluoroscopy while the filter for cine-angiography was 0.1 mm/1.0 mm Cu/Al [10]. Correspondingly, NRS group consists of procedures performed within 2 different Siemens X-ray system (Siemens Axiom Artis Zee Biplane MN, 2009 manufactured/2010 installed, and Siemens Axiom Artis Zeego, 2011 manufactured/2011 installed, Siemens Medical Systems, Erlangen, Germany) and 1 GE system (GE Innova IGS 520, 2015 manufactured/2016 installed, GE Healthcare; Little Chalfont, United Kingdom). Both the Siemens and GE X-ray systems have set fluoroscopy frame rates as 7.5 f/s and cine-angiography frame rates as 15 f/s. The Cu filter range from 0.1–0.3 mm for fluoroscopy while not implemented for cine-angiography in both Siemens systems. A maximum of 0.3 mm Cu filter was implemented for fluoroscopy and cine-angiography in GE system. All systems mentioned above received regular radiation dose report detection and correction.

### 2.3. Study Endpoints

The primary end point was the relative dose reduction of total air kerma. The value of air kerma, dose-area product (DAP), and fluoroscopy time were registered as indicated by the X-ray system. An efficiency index (EI) [11] was calculated by fluoroscopy time/total air kerma. The secondary endpoint was the incidence of 30-day major adverse cardiac and cerebrovascular events (MACCE), as defined by the composite of all-cause death, reinfarction, ischemia-driven target vessel revascularization, and stroke. Reinfarction was defined as the same as the reported article by Mehran et al. [12].

### 2.4. Statistical Analysis

The data were expressed as the mean ± SD for the continuous variables, and as frequencies for the categorical variables. The comparison of continuous variables was performed by the independent Student's *t*-test or the Mann-Whitney *U* test as appropriate. Statistical analysis of the categorical variables was performed using the Pearson chi-square or Fisher's exact test as appropriate. We used Cox proportional hazard models to estimate the impact of optimized radiation strategy to clinical outcomes adjusting for the differences in patient baseline and angiographic factors. *p* values were two-tailed, and *p* < 0.05 was considered statistically significant. The data were analyzed with SPSS v.20.0 statistical software (SPSS, version 20.0, Inc., Chicago, IL, USA).

## 3. Results

### 3.1. Baseline Characteristics

The baseline clinical characteristics of the patients are summarized in Table 1. It is apparent from this table that no significant differences were found between the 2 groups. The mean age of our cohort was 64.7 ± 11.9 years and body mass index was 24.0 ± 2.8 kg/m^2^. Anterior STEMI comprised 49.1% of the overall patients and 1.9% of all patients presented as cardiogenic shock. The 2 groups had a similar hemodynamic status, depicted by blood pressure, heart rate, and Killip class.

### 3.2. Angiographic and Procedural Characteristics

Details of angiographic and procedural characteristics are depicted in Table 2. A high rate of radial access was observed in both ORS group (97.4%) and NRS (93.7%) group (*p*=0.238). Multiple-vessel disease accounted for 70.6% of all patients, and non-culprit vessel PCI were implemented in 10 (4.7%) patients. There were also no significant differences for angiographic and procedural characteristics between 2 groups.

### 3.3. Outcomes

**Figure 2:**
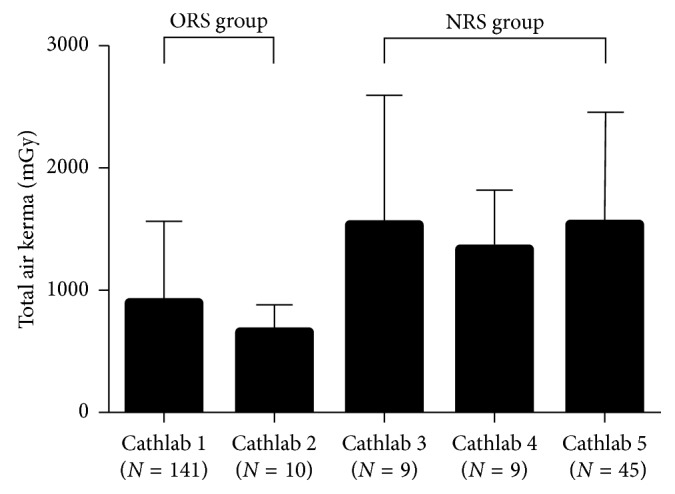
Radiation dose comparison among X-ray systems within groups. ORS, optimized radiation strategy; NRS, normal radiation strategy.

The differences of relevant radiation parameters between 2 groups are highlighted in Table 2. Fluoroscopy time was similar between 2 groups (20.1 ± 12.8 min versus 21.7 ± 17.4 min, *p*=0.439). The radiation dose reduction in ORS group was 40.9% for air kerma (901.2 ± 628.7 mGy versus 1524.0 ± 866.6 mGy, *p* < 0.001) and 43.5% for DAP (57.1 ± 40.7 Gycm^2^ versus 101.1 ± 59.4 Gycm^2^, *p* < 0.001). Meanwhile, EI increased by 55.0% (24.8 ± 9.5 min/Gy versus 16.0 ± 10.2 min/Gy, *p* < 0.001) in ORS group. Radiation dose comparison among X-ray systems within groups showed the total air kerma was comparable within NRS group, also for ORS group (Figure 2).

During 30-day period, no patient was lost to follow-up. Detailed clinical outcomes are described in Table 3. The cumulative incidence of all-cause death (2.0% versus 1.6%) and stroke (0.7% versus 0%) was similar between 2 groups. No reinfarction and ischemia-driven target vessel revascularization was observed. Two death were secondary to major bleeding while the other two secondary to cardiac shock. One stroke of intracranial bleeding was observed. After adjusting confounders, there were no significant differences of 30-day MACCE between 2 groups (2.0% versus 1.6%, adjusted hazard ratio: 0.7, 95% confidence interval: 0.1 to 8.6, *p*=0.772).

## 4. Discussion

We reported on the first study aimed to evaluate the efficacy and safety of an optimized radiation strategy versus normal radiation strategy in P-PCI for STEMI patients. The results showed a good combination of significant radiation dose reduction and similar 30-day outcomes from the optimized radiation strategy.

Reducing radiation exposure for both patients and interventional staff is a universal aim. Previous study reported some radiation reduction measures [6–9, 13]. which were all carried out in routine coronary angiography or selective PCI. There exist some differences between P-PCI and selective PCI. In view of the critical clinical situation of STEMI patients, evaluation of blood flow, culprit lesions, and thrombus burden should be clearly and rapidly accomplished. With regard to procedure details, P-PCI perform more thrombus aspiration and intracoronary injection. Moreover, high incidence of no reflow in P-PCI also demands the accurate judgment of the flow status. All these situations raise a claim for a good image quality. However, the evaluation of radiation protocol optimization for P-PCI has never been reported.

In the present study, significant radiation dose reduction was achieved in ORS group. This effect comes at no difference in fluoroscopy time, or contrast volume between 2 groups. As an indicator for radiation efficiency, the EI value in ORS group was obviously superior to NRS group. Thus, optimized radiation strategy demonstrated an obvious advantage of effectiveness.

For experienced operators, rather than trainees who may take longer fluoroscopy time to position catheters, cine-angiography usually occupied a bigger proportion of radiation dose. Consequently, it would be easier to achieve obvious radiation dose reduction by decreasing cine-angiography frame rate. Other than reduced frame rate, adoption of an additional Al filter, which has been reported to have additional radiation reduction effect [14], may play a very important role in dose reduction. Of course, in view of different installation years of serial X-ray systems, the radiation exposure secondary to the age of the hardware should also be taken into consideration.

In terms of radiation exposure control, how to strike a good balance between radiation dose reduction and image quality is always the key obstacle to overcome. Importantly, image quality should not only be evaluated by subjective visual feedback but also by objective clinical indicators. In the present study, the 30-day MACCE in 2 groups were both low and similar. Of course, longer follow-up time would be more powerful to evaluate the clinical outcomes.

This study had several limitations. First, this study was a retrospective, single institution design and included relatively small number of patients. However, it was the first study in this field. Second, during the period of this study, more P-PCI was carried out following the optimized radiation strategy. We should acknowledge the selection bias due to the nature of study design was present.

## 5. Conclusions

By investigating the efficacy and safety of an optimized radiation strategy in P-PCI for STEMI patients, we provided the primary evidence and experience in this field. The results of our study suggest that there would be considerable degree of radiation dose reduction in P-PCI for STEMI patients by applying proper optimized radiation strategy. It appears to be feasible and safe to carry out the optimized radiation strategy in P-PCI for STEMI patients.

## Figures and Tables

**Table 1:** Baseline clinical characteristics.

	ORS group (*n* = 151)	NRS group (*n* = 63)	*p* value
Gender, male	126 (83.4%)	55 (87.3%)	0.476
Age (years)	64.4 ± 12.2	65.2 ± 11.4	0.683
BMI (kg/m^2^)	24.0 ± 2.6	24.0 ± 3.2	0.906
Medical history			
Hypertension	99 (65.6%)	39 (61.9%)	0.610
Diabetes	44 (29.1%)	15 (23.8%)	0.427
Insulin-treated	5 (3.3%)	3 (4.8%)	0.696
Dyslipidemia	76 (50.3%)	26 (41.3%)	0.226
Smoking	90 (59.6%)	36 (57.1%)	0.739
Previous coronary intervention	43 (28.5%)	22 (34.9%)	0.350
Previous CABG	1 (0.7%)	1 (1.6%)	0.503
Clinical presentation			
Heart rate (beats/min)	77.8 ± 15.4	75.9 ± 13.9	0.399
Blood pressure (mm·Hg)			
Systolic blood pressure	120.7 ± 22.1	118.4 ± 20.4	0.489
Diastolic blood pressure	74.6 ± 14.2	71.7 ± 13.6	0.176
Cardiogenic shock on presentation	4 (2.6%)	0 (0%)	0.323
Infarct location			
Anterior	78 (51.7%)	27 (42.9%)	0.241
Not anterior	73 (48.3%)	36 (57.1%)	
Killip class			
I	139 (92.1%)	57 (90.5%)	0.705
II‒IV	12 (7.9%)	6 (9.5%)	
Left ventricular ejection fraction^*∗*^ (%)	52.4 ± 7.5	52.8 ± 8.9	0.747

Continuous data are presented as mean ± SD; categorical data are expressed as counts (percentage). ORS: optimized radiation strategy; NRS: normal radiation strategy; BMI: body mass index; CABG: coronary artery bypass grafting. ^*∗*^Value for 210 patients.

**Table 2:** Procedural and radiation dose characteristics.

	ORS group (*n* = 151)	NRS group (*n* = 63)	*p* value
Radial artery access	147 (97.4%)	59 (93.7%)	0.238
Multiple-vessel disease	109 (72.2%)	42 (66.7%)	0.419
Infarct related artery			
LM	1 (0.7%)	0 (0%)	0.628
LAD	77 (51.0%)	27 (42.9%)	
LCX	15 (9.9%)	8 (12.7%)	
RCA	58 (38.4%)	28 (44.4%)	
TIMI flow pre-PCI			
0	96 (63.6%)	45 (71.4%)	0.252
1	20 (13.2%)	3 (4.8%)	
2	12 (7.9%)	7 (11.1%)	
3	23 (15.2%)	8 (12.7%)	
Thrombus aspiration	108 (71.5%)	50 (79.4%)	0.234
Non-culprit vessel PCI	8 (5.3%)	2 (3.2%)	0.727
Stent number	1.5 ± 0.7	1.3 ± 0.8	0.241
GPI use			
During intervention	80 (53.0%)	31 (49.2%)	0.615
After intervention	40 (26.5%)	21 (33.3%)	0.312
Contrast volume (mL)	127.9 ± 43.8	122.4 ± 41.7	0.394
Successful PCI	151 (100%)	62 (98.4%)	0.294
Air kerma (mGy)	901.2 ± 628.7	1524.0 ± 866.6	<0.001
DAP (Gycm^2)^	57.1 ± 40.7	101.1 ± 59.4	<0.001
Fluoroscopy time (min)	20.1 ± 12.8	21.7 ± 17.4	0.439
EI (min/Gy)	24.8 ± 9.5	16.0 ± 10.2	<0.001

Continuous data are presented as mean ± SD; categorical data are expressed as counts (percentage). ORS: optimized radiation strategy; NRS: normal radiation strategy; LM: left main; LAD: left anterior descending artery; LCX: left circumflex artery; RCA: right coronary artery; TIMI: thrombolysis in myocardial infarction; PCI: percutaneous coronary intervention; GPI : GP IIb/IIIa inhibitor; DAP: dose-area product; EI: efficiency index.

**Table 3:** Clinical Outcomes at 30 days.

	ORS group (*n* = 151)	NRS group (*n* = 63)	Unadjusted HR (95% CI)	*p* value	Adjusted HR^*∗*^ (95% CI)	*p* value
MACCEs	3 (2.0%)	1 (1.6%)	1.3 (0.1–12.1)	0.844	0.7 (0.1–8.6)	0.772
All cause death	3 (2.0%)	1 (1.6%)	1.3 (0.1–12.1)	0.844	0.7 (0.1–8.7)	0.774
Reinfarction	0 (0%)	0 (0%)		NA		
Ischemia-driven target vessel revascularization	0 (0%)	0 (0%)		NA		
Stroke	1 (0.7%)	0 (0%)		1.000		

Categorical data are expressed as counts (percentage). ORS: optimized radiation strategy; NRS: normal radiation strategy; HR: hazard ratio; CI: confidence interval; MACCE: major adverse cardiac and cerebrovascular events; NA, not applicable. ^*∗*^Adjusted covariates included male, age, body mass index, diabetes, smoking, anterior infarction, multiple-vessel disease.

## Data Availability

The raw data that support the findings of this study are available only with a reasonable request to the corresponding author.
